# Pharmacist’s Intervention Considering the Prognosis for a Terminal Cancer Patient: A Case Report

**DOI:** 10.3390/pharmacy8040212

**Published:** 2020-11-11

**Authors:** Masahiro Okada, Kazuko Okazaki, Keisuke Kimura, Hiroki Sugihara, Fumiyoshi Murakami, Shinya Okamoto, Yoshinori Hoshino, Yuka Goto, Kengo Banshoya, Tadashi Onoda, Eisuke Takei, Shuso Takeda, Narumi Sugihara

**Affiliations:** 1 Department of Pharmacy Onomichi Municipal Hospital, Shintakayama, Onomichi, Hiroshima 3-1170-177, Japan; okada.masahiro@omhp.jp (M.O.); okazaki.kazuko@omhp.jp (K.O.); sugihara.hiroki@omhp.jp (H.S.); murakami.fumiyoahi@omhp.jp (F.M.); okamoto.shinya@omhp.jp (S.O.); hoshino.yoshinori@omhp.jp (Y.H.); gotou.yuka.2@omhp.jp (Y.G.); takei.eisuke@omhp.jp (E.T.); 2 Department of Surgery Onomichi Municipal Hospital, Shintakayama, Onomichi, Hiroshima 3-1170-177, Japan; kimura.keisuke@omhp.jp (K.K.); onoda.tadashi@omhp.jp (T.O.); 3 Faculty of Pharmacy and Pharmaceutical Sciences, Fukuyama University, Hiroshima 729-0292, Japan; kban@fukuyama-u.ac.jp (K.B.); s.takeda@fukuyama-u.ac.jp (S.T.)

**Keywords:** pharmacist intervention, prognosis, Palliative Prognostic Index, terminal cancer patient

## Abstract

Prognostic prediction has been reported to affect the decision of doctors and non-physician health care providers such as nurses, social workers, pastors, and hospice volunteers on the selection of appropriate medical interventions. This was a case of a 65-year-old woman who presented with a poor oral intake. The patient had a history of sigmoid colon cancer with abdominal wall metastasis and peritoneal dissemination. On the day of admission, nausea, anorexia, and malaise were noted, requiring immediate intervention. The patient’s prognosis was predicted using the Palliative Prognostic Index. The pharmacist suggested the use of dexamethasone tablets in order to alleviate the patient’s symptoms. Indeed, the administration of dexamethasone alleviated the symptoms of nausea, loss of appetite, and malaise. To the best of our knowledge, this is the first case report to demonstrate that prognosis prediction is important not only for other medical staff but also for pharmacists when deciding the need to initiate a treatment and continue such treatment, and when providing pharmacist interventions.

## 1. Introduction

Prognostic prediction has been reported to affect the decision of doctors and non-physician health care providers such as nurses, social workers, pastors, and hospice volunteers on appropriate medical interventions [1]. Morita et al. [2] reported that the long-term use of midazolam can increase the risk of developing drug tolerance; therefore, midazolam should only be used for patients with limited prognoses. Similarly, Morita et al. [3] reported that one liter or more of hydration per day could worsen peripheral edema, ascites, and pleural effusions in terminally ill cancer patients.

Traditionally, prognostic prediction has been based on the experience of clinicians. However, the development of objective prognostic prediction methods such as the Palliative Prognostic Index (PPI) [4] that can be used for terminally ill patients has made it possible for non-clinician medical staff to predict the patient’s prognosis. Baba et al. [5] reported that the PPI is simple and highly feasible. PPI without blood tests is also feasible for pharmacists. However, to the best of our knowledge, no study has reported that prognostic prediction can affect the pharmacists’ decision regarding the selection of appropriate medical interventions.

In this paper, we describe a case in which a pharmacist predicted the prognosis of a terminal cancer patient using the PPI and performed pharmacist interventions. Here, we report that prognosis prediction is important for pharmacists in the selection of treatment.

## 2. Case Presentation Section

### 2.1. Case Report

A 65-year-old woman presented with a poor oral intake. She had no significant past medical history. She had sigmoid colon cancer with abdominal wall metastasis and peritoneal dissemination. She had been treated with loxoprofen sodium hydrate and hydromorphone hydrochloride. She used hydromorphone hydrochloride, as needed, as treatment for pain, and domperidone, as needed, as treatment for nausea and vomiting.

### 2.2. Prognostic Predictive by PPI and Steroid Therapy

The five variables used to determine the PPI are as follows: Palliative Performance Scale (PPS), oral intake, dyspnea at rest, delirium, and edema [4]. The PPS [6], a modification of the Karnofsky Performance Scale, measures ambulation, activity, selfcare, intake, and level of consciousness. A PPI score of 4 or less predicts a survival of more than 6 weeks, while a PPI score of 6 or more predicts a survival less than 3 weeks [4]. Administration of corticosteroids has been reported to be effective for symptoms such as nausea, anorexia, and malaise in advanced cancer patients [7,8,9]. In Japan, Matsuo et al. [10] reported that steroid therapy is often initiated within the prognosis of 1 to 2 months.

### 2.3. Pharmacist Intervention

On the day of admission (Day 1), grade 3 nausea, anorexia, and malaise were observed using the Common Terminology Criteria for Adverse Events (CTCAE) version 5.0. Based on the severity of symptoms, the patient required immediate management to alleviate these symptoms.

Therefore, the prognosis of this patient was predicted using the PPI. The patient’s PPI was 3.5 (Table 1), and the prognosis was judged to be 1 to 2 months. Glare et al. [11] reported that the doctor’s prognosis was longer than the actual prognosis. In this case, as in the case of Glare et al., the doctor may have overestimated the prognosis. For the above reasons, the pharmacist suggested the use of oral dexamethasone in order to alleviate the patient’s symptoms. On Day 2, treatment with dexamethasone 2 mg/day was started.

The day after the administration of dexamethasone (Day 3), the severity of nausea decreased from Grade 3 to Grade 0–1, and the severity of anorexia and malaise decreased from Grade 3 to Grade 1 (Table 2). Based on the above results, the administration of dexamethasone was considered to alleviate the symptoms of nausea, loss of appetite, and malaise. She had a temporary mechanical ileus, but administration of dexamethasone 2 mg/day was effective for about 2 weeks for nausea, anorexia, and malaise. However, since the effect gradually disappeared, treatment with dexamethasone 4 mg/day was started on Day 23, but no effect was observed, and she died on Day 34.

The severity of symptoms was evaluated using the Common Terminology Criteria for Adverse Events version 5.0.

### 2.4. Ethical Consideration

This study was approved by the research ethics committee of Onomichi Municipal Hospital (approval number 20-10, 23 September 2020).

## 3. Discussion

Here, we report a case in which the patient’s nausea, anorexia, and malaise were alleviated by performing the pharmacist’s interventions based on the results of the prediction of the terminal cancer patient’s prognosis using the PPI. It is considered that the patient’s quality of life (QOL) improved by alleviating the symptoms of nausea, loss of appetite, and malaise. To the best of our knowledge, this is the first case report to show that prognosis prediction is important not only for other medical staff, but also for pharmacists when deciding on the necessity of initiating a treatment and continuing such treatment, and when performing pharmacist interventions.

Mendis et al. [12] reported that prognosis prediction of multidisciplinary team can assist treating teams to recognize and articulate prognosis, facilitate treatment decisions, and plan end-of-life care appropriately. It is expected that prognosis prediction of pharmacists contributes to prognosis prediction of multidisciplinary team.

In the future, it is expected that it will become more common for pharmacists to predict the prognosis and perform pharmacist interventions. Besides, various tools for predicting the prognosis of terminal cancer patients have been developed in addition to the PPI. Moreover, new prognostic prediction tools that can be used for performing the appropriate pharmacist interventions will be developed.

## 4. Conclusions

To the best of our knowledge, this is the first case report to show that prognosis prediction is important not only for other medical staff but also for pharmacists when deciding on the necessity of initiating treatments and continuing such treatments and performing pharmacist interventions.

## Figures and Tables

**Table 1:** Palliative Prognostic Index of our case.

Variables	Partial Score	Partial Score of Our Case
Palliative Performance Scale		2.5
10–20	4	
30–50	2.5	
≥60	0	
Oral intake		1
Mouthful or less	2.5	
Reduced but more than mouthful	1	
Normal	0	
Dyspnea at rest		0
Present	3.5	
Absent	0	
Delirium		0
Present	4	
Absent	0	
Edema		0
Present	1	
Absent	0	

**Table 2:** Subjective symptoms.

	Before (Day 1)	After (Day 3)
Nausea	Grade 3	Grade 0–1
Anorexia	Grade 3	Grade 1
Malaise	Grade 3	Grade 1
